# Characteristics and Treatment Outcomes of “Transfer Out” TB Patients after Crosschecking Registers at Four Hospitals of Tigray Regional State, Ethiopia: 2011-2015

**DOI:** 10.1155/2019/1761694

**Published:** 2019-06-24

**Authors:** Haftamu Hailekiros, Mahmud Abdulkader Mahmud, Alemayehu Bayray Kahsay

**Affiliations:** ^1^Mekelle University College of Health Sciences, Department of Microbiology and Immunology, 1871, Ethiopia; ^2^Mekelle University College of Health Sciences, School of Public Health, Department of Epidemiology, 1871, Ethiopia

## Abstract

Globally, transferring TB patients to another health unit for treatment continuation is common trend while posing challenges for proper treatment outcomes monitoring. National guidelines indicated the importance of incorporating the treatment outcomes of those cases by the transferring unit when performing annual cohort analysis. However, in most instances, this is not taken into account. This study was conducted to determine the characteristics and treatment outcomes of ‘transfer out' TB cases during the time period between 2011 and 2015 at four Hospitals of Tigray: Ethiopia. Initial data was extracted from TB treatment logbooks of transferring units using standardize checklist that were followed by a visit to each of the receiving units. The generated data were entered and analyzed using SPSS v. 22.0. Descriptive statistics were computed. P-value less than 0.05 were considered statistically significant. A total of 143 TB patients were transferred out during the specified time period whereas 73.4% (105/143) patients were traced and classified as “arrivals”. From these, more than three-quarters, 87.6% (92/105), of patients had a successful treatment outcome (5.7% cured and 81.9% completed treatment) while 13/105 (12.3%) had an unsuccessful outcome (2.8% defaulted, 5.7% died, 1.9% failed, and 1.9% transferred out). However, none of the transferring unit received and traced status of the cases. Therefore, regular monitoring is needed to improve the existing communication gap.

## 1. Introduction

Tuberculosis (TB) is worldwide public health problem, more importantly; its burden remains to be enormous in developing countries. In Ethiopia, the TB case notification and detection rate are improving over the years [1]. According to the Ethiopian national TB guideline, all patients either clinically judged or bacteriologically confirmed should start treatment without delay under Directly Observed Treatment-short course (DOTS) [2]. However, it is fairly common for TB patients to seek treatment services at health facility other than where they initially diagnosed [3].

A transfer out patient (TO) is a patient who has been transferred out at any time during treatment to continue treatment at another facility [4]. Such intracountry movement of patients, from one treatment unit to another, for various reasons poses challenge for effective implementation of TB control programs worldwide [5]. Reports depicted that there are substantial variation interims of the proportion of TO patients from one country to another; for instance, in Sub-Saharan Africa countries it ranges from 1% to 26% [6, 7], while higher rates, 31% to 66%, have been previously reported from Ethiopia [8–10]. A research study conducted in Gondor, Ethiopia, also showed that 42% of TB patients were transferred out between 2003 and 2008 from a University hospital to continue their treatment at nearby health units [9].

The National TB program (NTP) guidelines indicate the necessity of incorporating treatment outcome of transfer out TB patients and reporting regularly to the national surveillance system by the transferring unit, as this unit notified and initiated treatment for this patient. However, essentially this is not done in most settings [11–13]. Moreover, there is a research gap towards describing and determining the treatment outcomes of those cases nationally and locally. Therefore, our study was conducted to describe the characteristics and treatment outcomes of “transfer out” TB cases between 2011 and 2015 at four Hospitals of Tigray regional state, Ethiopia.

## 2. Materials and Methods

### 2.1. Study Design, Population, and Period

A five-year retrospective record review was conducted on transferred out TB patients from 2011 to 2015 who had TB treatment follow-up of one month and above in one of the hospitals and transferred out to another treatment centers.

### 2.2. Eligibility Criteria

Patients diagnosed for any form of TB and started treatment from September 2011 to December 2015 and were transferred out after one month of treatment initiation were included in to the study. TB patients with incomplete information were excluded from the study.

### 2.3. Data Collection Tools and Methods

Trained data collectors use standardized checklist extracted data from TB treatment logbooks of one comprehensive specialized hospital and three general hospitals. This was followed by a visit to each of the receiving treatment units for crosschecking and obtaining treatment outcome of each eligible patient. Full name and TB register codes were used as patient identifiers.

### 2.4. Data Quality Assurance

Quality was maintained throughout the study. Onsite training was provided for data collectors, before data collection was started. Medical register of the patients was checked for completeness and clinical history of the patients was taken carefully without missing any variable. Data was checked regularly for completeness and validity.

### 2.5. Data Analysis

Data were entered and analyzed using Statistical Package for Social Sciences (SPSS) software version 22.0. (IBM, USA). Descriptive statistics were computed. P-value less than 0.05 was considered statistically significant.

### 2.6. Ethical Considerations

The study protocol was evaluated and approved by the Research Ethics Review Committee of College of Health Sciences, Mekelle University. Cooperation letters were obtained from Tigray region health bureau. Permission was also obtained from TB clinic of each health facility.

## 3. Results

### 3.1. Characteristics of Transferred Out Patients

A total of 143 TB patients were transferred out to other health institutions from the selected hospitals for treatment continuation. From these, only 73.4% (105/143) patients were traced and classified as “arrivals”. The remaining 38 (26.8%) were classified as “non-arrivals.” The age of the patients varied from 1 to 70 years with mean age of 30.97±SD (14.88), and majority of them, 72 (50.3%), were in the age range of 19-34. As it is shown in Table 1, majority of the study participants were males 84 (58.7%), live in rural areas 73 (51%), and transferred to health centers 84 (58.7%) rather than hospitals.

As Table 2 shows, of the “arrivals”, most of the patients arrived in the receiving units within 8 days, 81 (77%). Moreover, 45 (76.2%) reported to health centers, and 51 (82.3%) were rural residents, 51 (83.6%) males, 40 (80%) extra pulmonary, and 37 (88.1%) HIV negative status.

### 3.2. Treatment Outcomes of Transferred Out TB Patients

#### 3.2.1. Category of Patients

With regard to type of TB, 74 (51.7%) of the patients were diagnosed as extra pulmonary TB (EPTB), 47(32.9%) smear negative PTB, and 22(15.4%) smear positive PTB. The proportion of EPTB was slightly higher among males 40 (47.6%) and HIV negative 42 (65.6%) individuals. The TB/HIV coinfection rate was 8(24.2%), 16(48.5 %), and 9(27.3%) among smear positive PTB, negative PTB, and EPTB, respectively (Table 3).

#### 3.2.2. Treatment Outcome

As it is shown in Table 4, treatment outcomes were analyzed for the 105 “arriving” patients. More than three-quarters, 87.6% (92/105), of patients had a successful treatment outcome (5.7% cured and 81.9% completed treatment) while 13/105 (12.3%) had an unsuccessful outcome (2.8% defaulted, 5.7% died, 1.9% failed, and 1.9% transferred out). For all transferred-out patients, feedback on treatment outcomes was not reported back to transferring unit by the receiving units.

## 4. Discussions

No patient should be lost when transferred out between treatment units; which has obvious benefits for treatment outcome [9, 14]. In our study, 73.4% of patients were traced while 26.5% of patients' identifier were not identified in the register of the designated receiving units. As compared to studies conducted in Gondor (12%), Laos (4%), and Afghanistan (10%), our study revealed higher rates of “non-arrivals” [9, 15, 16]. A similar reason with the study conducted in Gondor may also explain our findings. Likely, due to the traditional trend of self-referral to another preferred facility or the patient might have died before reaching to the designated unit. Accordingly, this may elucidate that the communication gap between healthcare providers and patients is yet a prevailing problem and this signifies the need for further indepth investigations [9, 17].

The current study showed that majority of the TO patients were rural residents (51%) and transferred to health centers (58.7%) plus diagnosed as EPTB (51.7%). This could be attributed to the expansion of TB treatment units in rural areas of Ethiopia, in addition, to the transferring units were largely served as diagnostic centers [9]. Transferring patient should travel with an adequate supply of oral anti-TB drugs and also arrive at the designated treatment unit on time so that prompt treatment follow up and care will be insured. [13]. In the current study, 77% of patients arrived on the specified treatment unit within 8 days. This result showed that there is need for integrated work among district TB officers, transferring and receiving units, as the transfer process took longer for 23% of the patients.

The Ethiopian NTP guideline indicates that the treatment outcome result of TO patients should be communicated back to the transferring unit [9]. However, in our study, none of the receiving units had communicated/reported back, and reasons for not communicating are unclear. A similar trend was reported by a study conducted in Gondor, Ethiopia [11]. Nevertheless, the objective of this study is not to explore the reason for reporting failures; this implies that there is poorly functioning feedback communication/reporting system [18].

Confirming treatment outcomes of TB patient is fundamental component of DOTs program [11]. In this study, 87.6% of transferred out TB patients had a successful treatment outcome (5.7% cured and 81.9% completed treatment). Slightly higher treatment success rate is observed in our study comparing to the study conducted in Gondor, Ethiopia (79%) [9], while another study conducted in Northeastern Ethiopia (86.2% -95%) revealed comparable results [19]. On the other hand, higher treatment success (94.8%) rate was reported by single study conducted at Northwest Ethiopia [20]. Though there appears few published works to evaluate treatment outcome of transfer out TB patients that has limited our scope to synthesis more our findings, the observed treatment outcome can be regarded as satisfactory. This success rate might be attributable to relatively lower retransferred rates (1.9%), failures (1.9%), default rate (2.8%), and death rate (5.7%) and also indications of progressively improving TB patients care and follow up interventions in the country.

### 4.1. Study Limitations

Due to retrospective nature of the study design and extended study period (5 years), it was challenging to ascertain all the information sought to be collected as a result of missing records owing to poor record keeping practices. On top of this, an enormous number of cases were excluded from the study due to the high rate of “non-arrivals”; these altogether might have influenced the results. Besides, we cannot generalize with these findings, as the study is limited to only two administrative districts in the regional state. Notwithstanding the limitations, the study identified the existing communication gaps, which therefore needs timely, and innovative and feasible communication strategies.

### 4.2. Conclusion and Recommendations

In conclusion, 73.4% of TO patients were traced and overall satisfactory treatment outcome rate (87.6%) was observed. However, 26.5% TO patients were not traced and the reason for “non- arrivals” is not clear. In addition, treatment outcome of arrivals is not communicated back or none of the transferring unit received and traced status of the cases. Thus, the results revealed an early caution wherein there is dire need to improve the data management strategy through effective monitoring and evaluation systems.

## Figures and Tables

**Table 1:** Characteristics of transferred-out patients at the selected health units, Tigray, Ethiopia, 2011–2015 (N=143).

Characteristics		Transfer out(N=143 ) Number (%)	Arrivals (N=105) Number (%)	Non arrivals (N=38) Number (%)
Sex	Male	84(58.7)	63(75)	21(25)
	Female	59(41.2)	42(71.2)	17(28.8)

Age (years)	≤ 18	26(18.2)	20(76.9)	6(23.1)
	19-34	72(50.3)	47(65.3)	25(34.7)
	35-54	28(19.6)	26(92.9)	2(7.1)
	≥ 55	17(11.9)	12(70.5)	5(29.4)
	*Total*	*143*	*105(73.4)*	*38(26.6)*

Category of TB	New	143(100)	105(73.4)	38(36.2)
	Retreatment	-	-	-

Type of TB	PTB/smear positive	22(15.4)	14(63.6)	8(36.4)
	PTB/smear negative	47(33)	40(85%)	7(15)
	EPTB	74(51.7)	51(68.9)	23(31.1)

HIV Status	Negative	64(44.7)	43(67.2)	21(32.8)
	Positive	33(23.1)	24(72.7)	9(27.3)
	Unknown	46(32.2)	38(82.6)	8(17.4)

Residency	Rural	73(51)	62(85)	11(15)
	Urban	62(43.4)	43(49.4)	19(30.6)
	Unknown	8(5.6)	0	8(100)

Transferred to	Hospital	59(41.3)	45(76.3)	14(23.7)
	Health center	84(58.7)	60(71.4)	24(28.6)

Year of registration	2011	35(24.4)	19(54.3)	16(45.7)
	2012	19(13.3)	11(57.9)	8(42.1)
	2013	29(20.3)	24(82.8)	5(17.2)
	2014	36(25.2)	31(86)	5(13.9)
	2015	24(16.8)	20(83.3)	4(16.7)
	*Total*	*143*	*105(73.4)*	*38(26.6)*

*Abbreviations. *HIV: human immune deficiency virus, TB: tuberculosis, PTB: pulmonary tuberculosis, and EPTB: extra pulmonary tuberculosis

**Table 2:** Characteristics of transferred-out patients with regard to time period between transfer out and transfer at the selected health units, Tigray, Ethiopia, 2011–2015 (N=104).

Characteristics		Time period between transfer out and transfer in			
		<8 days	8-30 days	>30 days	Total
Sex	Female	30(69.7)	8(19)	4(9.3)	42
	Male	51(83.6)	4(6.4)	7(11.5)	62

Age	≤ 18	19(90.5)	1(4.7)	1(4.7)	21
	19-34	31(67.4)	6(13)	9(19.5)	46
	35-54	20(80)	4(16)	1(4)	25
	≥ 55	11(91.7)	1(8.3)	0	12
	*Total*	81(77)	12(11.4)	11(10.6)	*104*

Residency	Rural	51(82.3)	7(11.3)	4(6.5)	62
	Urban	30(71.4)	5(11.9)	7(16.7)	42

HIV status	Positive	15(62.5)	6(25)	3(12.5)	24
	Negative	37(88.1)	1(2.4)	4(9.5)	42
	Unknown	29(76.3)	5(13.2)	4(10.5)	38

Type of TB	PTB /smear positive	10(71.4)	2(14.3)	2(14.3)	14
	PTB/ smear negative	31(77.5)	4(10)	5(12.5)	40
	EPTB	40(80)	6(12)	4(8)	50

Transferred to	Hospital	36(80)	5(11.1)	4(8.9)	45
	Health center	45(76.2)	7(11.9)	7(11.9)	59

*Abbreviations.* HIV: human immune deficiency virus, TB: tuberculosis, PTB: pulmonary tuberculosis, and EPTB: extra pulmonary tuberculosis.

**Table 3:** Characteristics of transferred out patients with regard to type of TB at the selected health units, Tigray, Ethiopia, 2011–2015 (N=143).

Characteristics		Type of TB			
		PTB/smear positive	PTB/smear negative	EPTB	Total
Sex	Female	5(8.5)	20(33.9)	34(57.6)	59(41.3)
	Male	17(20.2)	27(31.1)	40(47.6)	84(58.7)
	*Total*	22(15.4)	47(32.9)	74(51.7)	*143*

Age	≤ 18	2(7.7)	6(23.1)	18(69.2)	26(18.2)
	19-34	17(23.6)	24(33.3)	31(43.1)	72(50.3)
	35-54	1(3.6)	10(35.7)	17(60.7)	28(19.6)
	≥ 55	2(11.8)	7(41.2)	8(47.1)	17(11.9)
	*Total*	22(15.4)	47(32.9)	74(51.7)	*143*

Residency	Rural	7(9.6)	29(39.7)	37(50.7)	73(51)
	Urban	15(24.2)	18(29)	29(46.8)	62(43.4)
	Unknown	0	0	8(100)	8(5.6)
	*Total*	22(15.4)	47(32.9)	74(51.7)	*143*

HIV status	Positive	8(24.2)	16(48.5)	9(27.3)	33(23.)
	Negative	10(15.6)	12(18.6)	42(65.6)	64(44.8)
	Unknown	4(8.7)	19(41.3)	23(50)	46(32.2)
	*Total*	22(15.4)	47(32.9)	74(51.7)	*143*

Year of transfer out	2011	11(33.3)	3(9.1)	19(57.6)	33(23)
	2012	1(5.6)	7(38.9)	10(55.6)	18(12.6)
	2013	1(4.2)	10(41.7)	13(54.2)	24(16.8)
	2014	5(11.9)	21(50)	16(38.1)	42(29.4)
	2015	5(19.2)	5(19.2)	16(61.6)	26(18.2)
	*Total*	*23(16.1)*	*46(32.2)*	*74(51.7)*	*143*

*Abbreviations.* HIV: human immune deficiency virus, TB: tuberculosis, PTB: pulmonary tuberculosis, and EPTB: extra pulmonary tuberculosis.

**Table 4:** Treatment outcomes of transferred out TB patients who arrived at their receiving facility in Tigray, Ethiopia, 2011–2015 (*N* = 105).

Characteristics		Treatment outcome n(%)						
		Cured	Treatment completed	Default	Failure	Death	Transfer out	P-value
Sex	Male	6(9.5)	46(73)	3(4.7)	2(3)	4(6.3)	2(3)	0.062
	Female	0	41(95.3)	0	0	2(4.7)	0	

Age(years)	≤18	2(9.5)	18(85.7)	0	0	1(4.7)	0	
	19-34	4(8.5)	38(80.6)	1(2)	2(4.2)	1(2)	1(2)	
	35-54	0	19(73)	2(7.7)	0	4(15.4)	1(3.8)	
	≥55	0	12(100)	0	0	0	0	
	*Total*	*6(5.7)*	*87(82.8)*	*3((2.8)*	*2(1.9)*	*6(5.7)*	*2(1.9)*	

Residency	Urban	4(9.3)	36(8.4)	1(2.3)	0	1(2.3)	1(2.3)	0.461
	Rural	2(3.2)	51(81)	2(3.2)	2(3.2)	5(7.9)	1(1.6)	

Category of TB	New	6(5.7)	86(82)	3(2.8)	2(1.9)	6(5.7)	2(1.9)	
	Retreatment	-	-	-	-	-	-	

Type of TB	PTB/Smear positive	6(42.8)	6(42.8)	0	1(7.1)	1(7.1)	0	0.062
	PTB/Smear negative	0	35(85.4)	1(2.4)	1(2.4)	2(4.9)	2(4.9)	
	EPTB	0	46(90.2)	2(3.9)	0	3(5.9)	0	

HIV status	Negative	4(9.3)	36(83.7)	2(4.6)	0	1(2.3)	0	0.156
	Positive	1(4)	19(76)	0	0	4(19)	1(4)	
	Unknown	1(2.6)	32(84.2)	1(2.6)	2(5.2)	1(2.6)	1(2.6)	

Time period b/n transfer-out & transfer- in	<8 days	5(6.1)	70(85.4)	2(2.4)	1(1.2)	3(3.7)	1(1.2)	0.164
	8-30 days	0	9(75)	0	0	3(25)	0	
	>30 days	1(9.1)	9(81.8)	0	1(9.1)	0	0	

Treatment center	Hospital	3(6.5)	38(84.4)	1(2.2)	0	3(6.5)	0	0.641
	Health center	3(4.9)	49(80.3)	2(3.3)	2(3.3)	3(4.9)	2(3.3)	

*Abbreviations. *HIV: human immune deficiency virus, TB: tuberculosis, PTB: pulmonary tuberculosis, and EPTB: extra pulmonary tuberculosis.

## Data Availability

The data used to support the findings of this study are available from the corresponding author upon request.
